# The length of FOXE1 polyalanine tract in congenital hypothyroidism: Evidence for a pathogenic role from familial, molecular and cohort studies

**DOI:** 10.3389/fendo.2023.1127312

**Published:** 2023-03-16

**Authors:** Elisa Stellaria Grassi, Giuditta Rurale, Tiziana de Filippis, Davide Gentilini, Erika Carbone, Francesca Coscia, Sarah Uraghi, Martyn Bullock, Roderick J. Clifton-Bligh, Abhinav K. Gupta, Luca Persani

**Affiliations:** ^1^ Department of Biotechnology and Translational Medicine, University of Milan, Milan, Italy; ^2^ Laboratory of Endocrine and Metabolic Research, Istituto di Ricovero e Cura a Carattere Scientifico (IRCCS) Istituto Auxologico Italiano, Milan, Italy; ^3^ Department of Brain and Behavioral Sciences, University of Pavia, Pavia, Italy; ^4^ Istituto Auxologico Italiano, Istituto di Ricovero e Cura a Carattere Scientifico (IRCCS), Bioinformatics and Statistical Genomics Unit, Milano, Italy; ^5^ Structural Biology Centre, Human Technopole, Milano, Italy; ^6^ Department of Health Science, University of Milan, Milan, Italy; ^7^ Cancer Genetics Laboratory, Kolling Institute, Faculty of Medicine and Health, The University of Sydney, Sydney, NSW, Australia; ^8^ Department of Endocrinology, Royal North Shore Hospital, St Leonards, NSW, Australia; ^9^ Department of Diabetes and Endocrine Sciences, CK Birla Hospitals, Jaipur, Rajasthan, India

**Keywords:** FOXE1, congenital hypothyroidism, athyreosis, polyalanine tracts, forkhead transcription factor, next generation sequencing

## Abstract

**Introduction:**

*FOXE1* is required for thyroid function and its homozygous mutations cause a rare syndromic form of congenital hypothyroidism (CH). *FOXE1* has a polymorphic polyalanine tract whose involvement in thyroid pathology is controversial. Starting from genetic studies in a CH family, we explored the functional role and involvement of *FOXE1* variations in a large CH population.

**Methods:**

We applied NGS screening to a large CH family and a cohort of 1752 individuals and validated these results by *in silico* modeling and *in vitro* experiments.

**Results:**

A new heterozygous *FOXE1* variant segregated with 14-Alanine tract homozygosity in 5 CH siblings with athyreosis. The p.L107V variant demonstrated to significantly reduce the FOXE1 transcriptional activity. The 14-Alanine-FOXE1 displayed altered subcellular localization and significantly impaired synergy with other transcription factors, when compared with the more common 16-Alanine-FOXE1. The CH group with thyroid dysgenesis was largely and significantly enriched with the 14-Alanine-*FOXE1* homozygosity.

**Discussion:**

We provide new evidence that disentangle the pathophysiological role of FOXE1 polyalanine tract, thereby significantly broadening the perspective on the role of *FOXE1* in the complex pathogenesis of CH. FOXE1 should be therefore added to the group of polyalanine disease-associated transcription factors.

## 1 Introduction

Congenital hypothyroidism (CH) is one of the most common preventable causes of intellectual disability (1). In the last two decades, the refinement of the neonatal screenings toward lower blood TSH cutoffs brought the estimated incidence of CH from 1:4000 to 1:2000 (1–4), with significant variations depending on geographic location, ethnicity, gender and pregnancy conditions (3). CH can be due to either defects in thyroid organogenesis, collectively called thyroid dysgenesis (TD), or in thyroid hormonogenesis, called dyshormonogenic defects, in the presence of a normal or enlarged gland *in situ* (GIS) (3, 5). Consistent with such heterogeneity, CH was considered a puzzle of monogenic diseases, but the molecular mechanisms responsible for CH, particularly in TD, are still largely undefined (5, 6). Interestingly, the evidence of frequent oligogenic defects (6, 7) and the increased risk for CH in particular conditions (3, 4) proposed a more complex pathogenesis for CH, which might also explain the sporadic appearance of the disease in one family (5–8).

*Forkhead Box E*1 (*FOXE1)* is one of the candidate genes for TD, but its involvement in the CH pathogenesis has not been yet clarified. Homozygous point mutations in the DNA-binding domain (DBD) of this transcription factor are reported to be responsible for the rare Bamforth-Lazarus syndrome characterized by TD together with bifid epiglottis, cleft palate and spiky hair (9–11). FOXE1 is composed by highly conserved DBD, followed by a polyalanine (poly-Ala) tract of variable length and by a C-terminal disordered region of yet unknown significance (11, 12). During development and adult life, FOXE1 may act both as a pioneer transcription factor and as a co-regulator of the actions of other transcription factors through its DBD, poly-Ala domain or *via* its unstructured portion (13, 14). In particular, FOXE1 together with HEEX, NKX2.1 and PAX8 constitute a finely tuned system that regulates the expression of genes involved in thyrocyte precursors migration, differentiation and finally thyroid hormone production (13, 14).

Interestingly, some studies suggested a possible predisposing role for the FOXE1 poly-Ala tract in CH, but this is still highly controversial due to the limited size of the population studies or variable experimental conditions (12, 15–19).

Here, we bring new evidence about the role of *FOXE1* in thyroid pathogenesis. Our data, obtained from Next Generation Sequencing (NGS) genetic screening of a large family and of a large CH cohort, *in silico* modelling and *in vitro* experiments, indicate that variations in the *FOXE1* poly-Ala tract length may affect both its own transcriptional activity as well as its synergic action with PAX8 and NKX2-1 on the thyroglobulin (*TG*) promoter.

## 2 Materials and methods

### 2.1 Enrolment of the family

**Figure 1:**
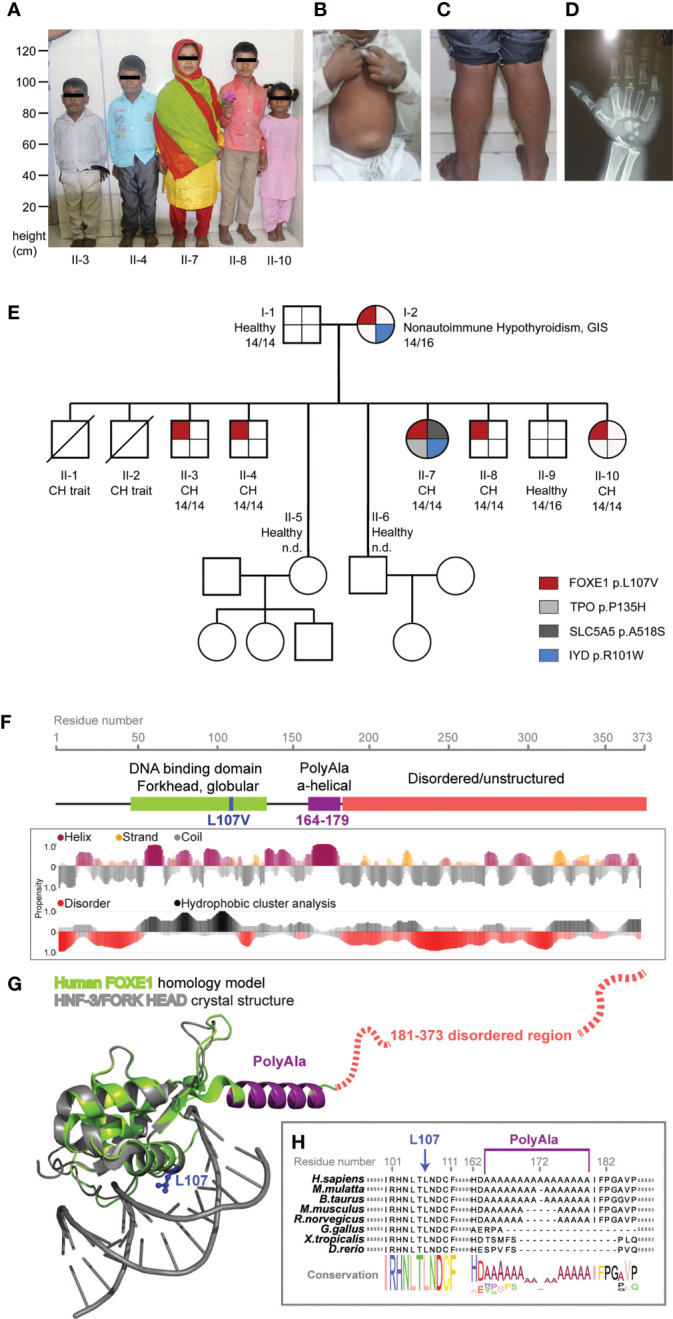
*FOXE1* variants segregation in the family and protein structural modeling. **(A)** picture of the five affected siblings showing severe short stature, puffy facies with dry skin, sparse fragile hair, lateral madarosis and short round nose. **(B)** umbilical hernia in patient II-3. **(C)** calf muscle pseudo-hypertrophy in patient II-4. **(D)** X ray of left hand revealing delayed bone age and epiphyseal dysplasia in patient II-7. **(E)** family tree showing *FOXE1* and other genetic variants co-segregation with CH (14/14, Ala-14/14 FOXE1; 14/16, Ala-14/16 FOXE1; 16/16, Ala-16/16 FOXE1; n.d., not determined). **(F)** linear representation of FOXE1 sequence and local secondary structure prediction adopted by the FOXE1 aminoacidic residues calculated with the software FELLS. The plot indicates the probability of a defined secondary structure or disordered coiled to be adopted per sequence residue. **(G)** AlphaFold *in silico* model of the human FOXE1 structured region (residues 56-181), superimposed with the experimental crystal structure of a homologue transcription factor bound to DNA (HNF-3/forkhead, PDB ID: 1VTN). FOXE1 model shows a conserved DNA binding domain (green), followed by a poly-alanine alpha-helical region (purple) and by and unstructured disordered region (red dotted line). The relative position of L107 residue and polyalanine tract in respect to DNA binding suggest proximity but not apparent direct binding to DNA. **(H)** FOXE1 alignment showing the highly conserved L107 residue in the DNA binding domain and the relatively late evolutionary emergence of the polyalanine tract.

The family was enrolled by AKG who made the diagnosis and collected the clinical and biochemical data (**Table 1**). AKG obtained informed consent of the parents for genetic studies and publication of the family pictures (**Figures 1A–F**).

**Table 1:** Clinical and laboratory data in the Indian family.

	I-1 Father	I-2 Mother	II-3 CH	II-4 CH	II-7 CH	II-8 CH	II-9 normal	II-10 CH
**Sex (M/F)**	M	F	M	M	F	M	M	F
**Age (years)**	60	53	25	22	19	17	12	10
**Weight (kg)**	–	–	20	24	37	28	35	15
**Height (cm)**	–	–	100	113	122	124	142	98
**Serum TSH at CH diagnosis** **(nv: 0.34-4.25 mU/L)**	2.60	18.64	424.0	478.0	470.0	318.0	2.32	398.0
**Total T4 at CH diagnosis** **(nv: 70-150 nmol/L)**	–	–	21.5	15.8	18.4	25.5	–	25.3
**Total T3 at CH diagnosis** **(nv: 1.2-2.1 nmol/L)**	–	–	<0.1	<0.1	<0.1	<0.1	–	<0.1
**Free T4** **(nv: 8-16 pmol/L)**	11.6	8.2	–	–	–	–	12.2	–
**Free T3** **(nv: 3.7-6.5 pmol/L)**	5.2	4.0	–	–	–	–	6.1	–
**Tg at CH diagnosis** **(nv: 13-118 μg/L)**	–	–	5.3	3.2	4.3	5.8	–	6.4
**Anti-TPO Abs** **(nv: <35klU/l)**	<10	<10	<10	<10	<10	<10	<10	<10
**Anti-Tg Abs** **(nv: <40klU/l)**	–	–	<15	<15	<15	<15	–	<15
**Thyroid US and scintiscan**	–	GIS	Athy	Athy	Athy	Athy	–	Athy

CH, congenital hypothyroidism; nv, normal values; Tg, thyroglobulin; Athy, athyreosis; GIS, gland-in-situ.

### 2.2 NGS patients database

Our database was composed of 1752 individuals analysed in our laboratory by NGS from 2015 to 2019. The CH patients were 299, 224 of which with complete clinical information available that allowed classification as GIS (66%) or TD (34%, of which 34% athyreosis, 39% ectopy, 27% hypoplasia); 1453 individuals without history and biochemical evidence of thyroid disease were used as control group. Written informed consent was obtained from all participants recruited in this study (Ethic Committee of Istituto Auxologico Italiano approvals 05C002_2010 and 05M001_2012).

### 2.3 Nextera rapid capture enrichment

A custom NGS panel covering all exons and adjacent intronic regions of the *DUOX2*, *DUOXA2*, *FOXE1*, *GLIS3*, *IYD*, *JAG1*, *NKX2-1*, *NKX2-5*, *PAX8*, *SLC26A4, SLC5A5*, *TG*, *TPO* and *TSHR* genes was designed using the GenomeStudio software (Illumina, San Diego, CA). NGS procedures and data analysis were performed as previously described (7). The total coverage of the target genes by the designed amplicons was 94%; these regions were covered at least by 20x. The uncovered sequences were amplified and sequenced by Sanger sequencing Big Dye Terminator Kit (Life Technologies) or by Nextera XT DNA Library Preparation kit (Illumina, San Diego, CA).

### 2.4 Bioinformatics analyses

NGS sequence data were aligned to the human reference genome (UCSC hg 19) and processed with MiSeq Reporter (Illumina) and wANNOVAR software. The visual inspection of the mapped data was performed using the Integrated Genomics Viewer 2.3 software (IGV; Broad Institute, Cambridge, MA, USA). The variants with the minor allele frequency (MAF) > 0.1% and annotated as benign in public or licensed databases (NCBI-dbSNP, NCBI-CliVar, Ensembl, GnomAD, ExAC Browser, NHLBI GO Exome Sequencing Project and HGMD professional, CLINVITAE) were excluded.

The different variants were analyzed according to ACMG/AMP 2015 guidelines (20) by the Varsome (https://varsome.com/) (21) and wIntervar (http://wintervar.wglab.org/) (22) bioinformatics tools for clinical interpretation of genetic variants together with the review of the scientific literature and 12 predictive software for interpretation of nonsynonymous variants (SIFT, Polyphen2_HDIV, Polyphen2_HVAR, LRT, MutationTaster, MutationAssessor, FATHMM, PROVEAN, MetaSVM, MetaLR, M-CAP, Fathmm-MKL).

### 2.5 Structural modelling

FOXE1 local secondary structure prediction was calculated with the software FELLS (23). The resulting plot shown in **Figure 1** indicates the probability of a defined secondary structure to be adopted by protein sequence residue.

Human FOXE1 structured region (residues 56-181) *in silico* model was obtained with AlphaFold (24) and superimposed to the experimental crystal structure of a homologue transcription factor bound to DNA (HNF-3/forkhead, PDB ID: 1VTN).

### 2.6 Cell line, mutagenesis and transfections

HEK293 (RRID : CVCL_0045) cells were grown in DMEM while NTHY-ORI 3-1 cells (RRID : CVCL_2659) were grown in RPMI-1640 medium, both supplemented with 10% fetal bovine serum and 1:100 penicillin-streptomycin (Life Technologies). They were cultured at 37°C in humidified 5% CO_2_ environment and were routinely tested for Mycoplasma.

p3xFlag-CMV-7.1 *FOXE1* cDNA expression vectors were previously described (25), p.Leu107Val variant was introduced by site-directed mutagenesis with primers designed with NEBaseChanger version v1.3.0 software (Forward: 5’- CAACCTCACAGTCAACGACTGC, Reverse: 5’- TGGCGGATGCTGTTCTGC). The mutagenesis was performed using NEB’s Q5 Hot Start High-Fidelity Polymerase (cat. no. M0493S), the reaction was supplemented with 20% Q-solution (Qiagen) to facilitate amplification of FOXE1’s GC-rich sequence.

Transient transfection was performed with Lipofectamine™ 2000 Transfection Reagent (Life Technologies) following manufacturer’s instructions. All experiments were performed 24 hours post-transfection.

### 2.7 Western blotting

Cells were lysed with RIPA buffer (Sigma) supplemented with Complete Mini protease and phosphatase inhibitor cocktails (Roche). 5 μg of proteins were loaded on NuPAGE 4–12% Bis-Tris gel and transferred on Nitrocellulose membranes with iBlot system (Life Technologies).

After blocking in 5% milk-TBST, membranes were incubated overnight with primary antibodies anti-FLAG (M2, Sigma RRID: AB_259529) and anti-GAPDH (sc-25778, Santa Cruz Biotechnology RRID : AB_10167668).

After 1 hour incubation in the appropriate HRP conjugated secondary antibodies (Merck Millipore), detection was performed with ECL Star (Euroclone) with Azure Biosystem C400 camera. Densitometric quantification was performed with FIJI (RRID : SCR_002285) (26).

### 2.8 Immunofluorescence and confocal microscopy

Samples were fixed in 4% PFA for 10 minutes and permeabilized with 0.3% Triton-X/PBS for 5 minutes. After blocking with 5% donkey serum/PBS at RT for 20 minutes, samples were incubated at 37°C for 1 hour with 1:100 anti-FLAG M2 mouse monoclonal antibody (Sigma). Samples were incubated for 1 hour with 1:500 AlexaFluor-488 secondary antibody (Thermo-Fisher). Samples were mounted on microscope slides with Vectashield Hard Set with DAPI (DAKO). Images were acquired with Nikon EclipseTi-E inverted microscope with IMA10X Argon-ion laser System (Melles Griot). For FOXE1 expression pattern, DAPI was used to determine ROI for further analysis and batch level thresholding was applied to the green channel originating a grayscale image utilized for further analysis. Each transfected cell was manually assigned to one of the 3 nuclear signal pattern categories. The analysis was performed in parallel by three independent operators (ESG, TdF and GR). All images processing and analysis were performed with FIJI (26).

### 2.9 Luciferase assay

FOXE1 activity was measured with the Dual-Luciferase Reporter Assay System (Promega).

**Figure 2:**
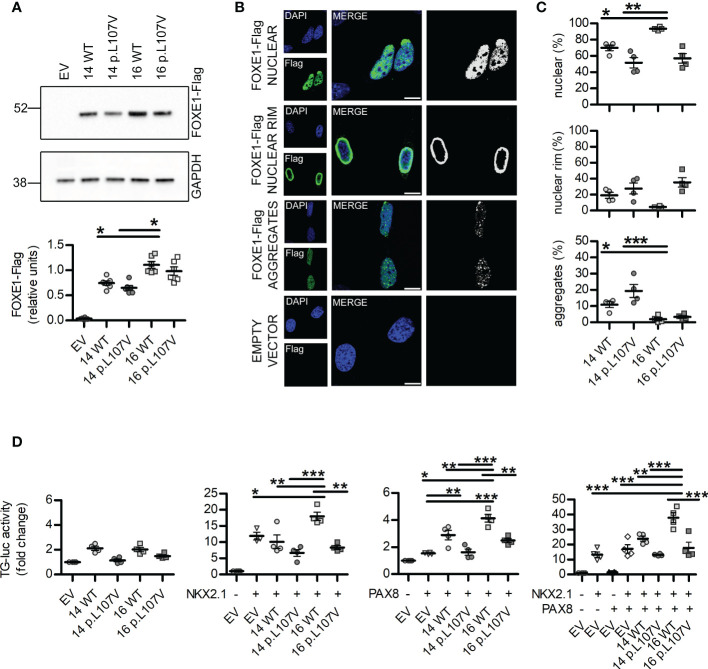
*In vitro* studies of FOXE1 variants reveal different subcellular localization and transcriptional activity. **(A)** representative images and quantification of western blot experiments showing FOXE1 variants expression levels in NTHY-ORI cells (n=6). **(B)** confocal microscopy images representative of the different FOXE1 nuclear morphologies in NTHY-ORI cells and corresponding signal thresholding images. **(C)** relative quantification of FOXE1 variants nuclear morphologies in NTHY-ORI cells (n=4; 1711 nuclei analyzed, of which 16 WT 295, 14 WT 258, 16 p.L107V 269, 14 p.L107V 295); scalebars 25µm. **(D)** functional assays showing FOXE1 variants activity with TG-luc reporter when *FOXE1* was transfected alone, with *NKX2.1*, *PAX8* or all together (n=4). EV, Empty vector. Statistical significance was determined with one-way ANOVA followed by Bonferroni *post-hoc* test **(A, D)** or Kruskal-Wallis **(C)**. *p<0.05, **p<0.01, ***p<0.001.

250 ng of Luciferase reporter plasmid TG-Luc (27) were co-transfected with 80 ng of pRL-TK Renilla construct (Promega, Madison, Wisc., USA), 250 ng of *FOXE1* constructs, and/or 250 ng of NKX2.1, PAX8 (27), empty vector as indicated in **Figure 2** and **Supplementary Figure 1**. 24 hours post transfection luminescence was measured with Dual Luciferase kit (Promega) with Fluoroskan Ascent FL multiplate reader.

### 2.10 Statistical analysis

All experiments were independently repeated at least three times, as indicated in the figure legends; data represent mean ± SEM. All analyses were performed with software R version 4.1.2. Contingency was evaluated by Chi-square, Chi-square test for trend and Fisher’s exact test. For *in vitro* experiments, after normal distribution and variance similarity evaluation by Bartlett’s test, one-way ANOVA followed by Bonferroni *post-hoc* test or Kruskal-Wallis test were applied.

## 3 Results

### 3.1 Clinical data

Five siblings of a large family presented to the endocrinology outpatient clinic with complaints of lethargy, constipation, short stature and intellectual disability. All children were born from a non-consanguineous marriage with uneventful pregnancies and deliveries. All the affected siblings were reported to have had feeding difficulties and delayed developmental milestones. Family history revealed premature death of 2 additional siblings at the ages of 2 and 32 years for infectious diseases, but with complaints similar to the CH affected siblings. The remaining 3 out of the 10 siblings had no stigmata of thyroid disease.

On general examination all the affected siblings had typical signs of untreated severe CH (**Figures 1A–D**). The five siblings presented extremely short stature, typical puffy hypothyroid facies with cold dry skin, sparse fragile hair, lateral madarosis, short round nose (**Figure 1A**), umbilical hernia (**Figure 1B**), calf muscle pseudo-hypertrophy (**Figure 1C**) macroglossia, myxedema, hoarseness of voice, delayed relaxation of deep tendon reflexes, pseudomyotonia and bradycardia. X ray examination revealed delayed bone age and epiphyseal dysplasia (**Figure 1D**). Thyroid was not detectable at neck palpation or ultrasonography as well as on neck/mediastinum ^99^Tc scintiscan. Biochemical investigations revealed extremely high level of TSH along with markedly low T4, T3 and Tg. Anti- thyroid peroxidase (Anti-TPO) and Anti-TG antibodies were negative (**Table 1**). Lab tests also showed normal hemogram, kidney and liver function. Electrolytes, LH, FSH and Prolactin were in the normal range for age.

Maternal laboratory testing revealed high TSH, negative anti-thyroid antibodies and normally located thyroid gland on ultrasonography. Father’s thyroid function tests were all normal.

All five siblings were diagnosed with severe CH associated with athyreosis and started on thyroxine replacement and dose titrated by periodic laboratory investigation.

### 3.2 NGS sequencing analysis revealed co-segregation of the CH phenotype with FOXE1 variants

The NGS sequencing of all available members revealed genetic alterations only in the affected patients: the mother carried a *IYD* variant (NC_000006.12(NM_203395.3):c.301C>T, rs121918138) that was transmitted to only one affected daughter. This same patient had two additional *de novo* variants in *SLC5A5* (NC_000019.10(NM_000453.3):c.1552G>T, rs147583297) and *TPO* (NC_000002.12(NM_000547.6):c.404C>A, rs61758083) (**Figure 1E**). The *IYD* variant had previously been described (28) and classified as pathogenic while the ones identified in *TPO* and *SLC5A5* are rare variants annotated as of unknown significance according to the American College of Medical Genetics and Genomics (ACMG) standards and guidelines (20) (**Supplementary Table 1**).

The mother and the five affected siblings were all found to carry a novel heterozygous *FOXE1* variant (**Figure 1E**) [NC_000009.12(NM_004473.4):c.319C>G, p.Leu107Val].

A subsequent in-depth analysis of the *FOXE1* gene revealed an association between the p.Leu107Val variant, the polyalanine region length of 14 alanines (Ala-14) and the CH phenotype in the family. The homozygous Ala-14 and the heterozygous p.Leu107Val *FOXE1* variants were present in all the five siblings with athyreosis. The hypothyroid mother carried the Ala-14/p.Leu107Val combined with the Ala-16 wild-type allele, while the euthyroid father carried the homozygous Ala-14 *FOXE1* but no additional variants in the CH candidate genes.

Moreover all the family members carried the SNP (NC_000009.12:g.97786731A>G rs7850258) in homozygosity, previously described as predisposing factor for CH (29).

### 3.3 *In silico* analysis suggests a possible effect of the p.Leu107Val and the Ala-14 *FOXE1* variants

I*n silico* predictions classified the p.Leu107Val variant as possibly pathogenic (**Supplementary Table 1**).

Using AlphaFold (24) we predicted the structure of FOXE1 (**Figures 1F–H**) and superimposed it with the experimental structure of a transcription factor of the same Forkhead family, bound to DNA (PDB ID: 1VTN). In the predicted FOXE1 model by comparison to the reference structure bound to a short linear DNA fragment, the highly conserved L107 appears located in a hydrophophic pocket relatively distant (about 7 Å) from DNA to be directly involved in its binding, as also the alpha-helical polyalanine tract. The same L107 structural position is occupied by a phenylalanine in the reference structure within a highly hydrophobic protein region. We hypothesize that the replacement of leucine with a similarly hydrophobic but shorter valine side chain, could potentially affect the folding of FOXE1, perhaps destabilizing it locally and impacting the transcriptional activity (**Figures 1F–H**). These alterations may become more pronounced in combination with variations of the polyAla tract in proximity of the DBD. Indeed, it has been observed that the amplifications of polyalanine tracts that are acquired late in evolution as *FOXE1* ones (**Figure 1H**) may have a fine, yet unclear transcriptional regulatory role (30, 31).

It has to be considered that many pioneer transcription factors as FOXE1 bind to non-linear DNA on nucleosomes and have large disordered regions. Our analysis is limited to short DNA linear fragments present in the Protein Data Bank and we don’t consider the highly C-terminal disordered region for which is impossible to reliably predict. Nonetheless, the current *in silico* analysis is important to highlight the non-obvious and complex molecular role of Poly-Ala tracts and L107V variants, which deserve finer molecular investigations in the future.

### 3.4 *In vitro* studies revealed negative effects of the p.Leu107Val and the Ala-14 *FOXE1* variants

We moved to *in vitro* experiments to evaluate possible alterations in FOXE1 expression and functionality. We performed transient transfection experiments on HEK293 and NTHY-ORI 3-1 cells with *FOXE1* either with Ala-14 or Ala-16, each one with (14 p.L107V, 16 p.L107V) or without (14 WT, 16 WT) the p.Leu107Val variant.

Western blotting experiments did not reveal variations among the different FOXE1 variants in HEK cells (**Supplementary Figure 1**) but showed a reduced expression of the Ala-14 FOXE1s when compared to the Ala-16 ones in the thyrocyte-derived NTHY-ORI cells (**Figure 2A**). These differences among the two cell lines are probably due to their different origins. NTHY-ORI are the only available cell line of immortalized human thyrocytes and may provide a more accurate model for thyroid transcription factors studies. Though they exhibit a partial loss of differentiation due to adherent cell culturing conditions, they possess the adequate biological machinery for FOXE1 expression and functionality and for this reason are probably more sensitive than HEK cells to FOXE1 alterations.

Confocal microscopy experiments indicated that the length of the polyalanine tract and the presence of p.Leu107Val variant can influence FOXE1 nuclear localization. In all the different FOXE conditions we identified three main different patterns, evenly diffuse nuclear signal (nuclear), uneven nuclear signal with significantly higher intensity at nuclear rim (nuclear rim), and the presence of nuclear puncta aggregates (aggregates) (**Figures 2B, C**, **Supplementary Figures 1B, C**, **2**).

The quantification of the different patterns revealed that while the Ala-16 WT protein has significantly higher proportion of cell that display an evenly diffuse nuclear pattern, the Ala-14 WT is more prone to form nuclear aggregates. The presence of the p.Leu107Val variant significantly reduced the percentage of cells with even nuclear signal while increasing the frequency of nuclear aggregates and nuclear rim in both poly-Ala backgrounds (**Figures 2B, C**, **Supplementary Figures 1B, C**).

Functional assays performed in NTHY-ORI cells revealed that the different FOXE1 variants have variable activities on the TG promoter (**Figure 2D**). First of all, when FOXE1 is expressed alone, the Ala-14 and Ala-16 have similar activity, and this is negatively affected by the presence of the p.Leu107Val variant. As during thyroid development and adult life FOXE1 is concomitantly expressed with PAX8 and NKX2.1 and it is expected to modulate their transcriptional activity (13), we performed different co-transfection experiments. Under these conditions, Ala-14 and Ala-16 FOXE1s have significantly different activities. In particular, only the Ala-16 FOXE1 significantly enhances NKX2.1 transcriptional activity, while PAX8 can be induced by both Ala-16 and Ala-14 FOXE1s, although the latter with significantly lower efficiency (**Figure 2D**). Moreover, when the three transcriptional factors are co-expressed, a significantly higher activity is detected only in the presence of the Ala-16 FOXE1 (**Figure 2D**). The introduction of the p.Leu107Val variant decreased the transcriptional activity in all the different experimental settings (**Figure 2D**).

Altogether these data indicate that both the presence of 14 alanines and p.Leu107Val variant in the DBD may negatively affect FOXE1 expression patterns and functionality, further supporting their role in the development of congenital hypothyroidism.

### 3.5 FOXE1 heterozygous variants and polyalanine region role in CH predisposition

We then evaluated the distribution of the different poly-Ala *FOXE1* tracts in 299 CH patients and 1453 controls.

**Figure 3:**
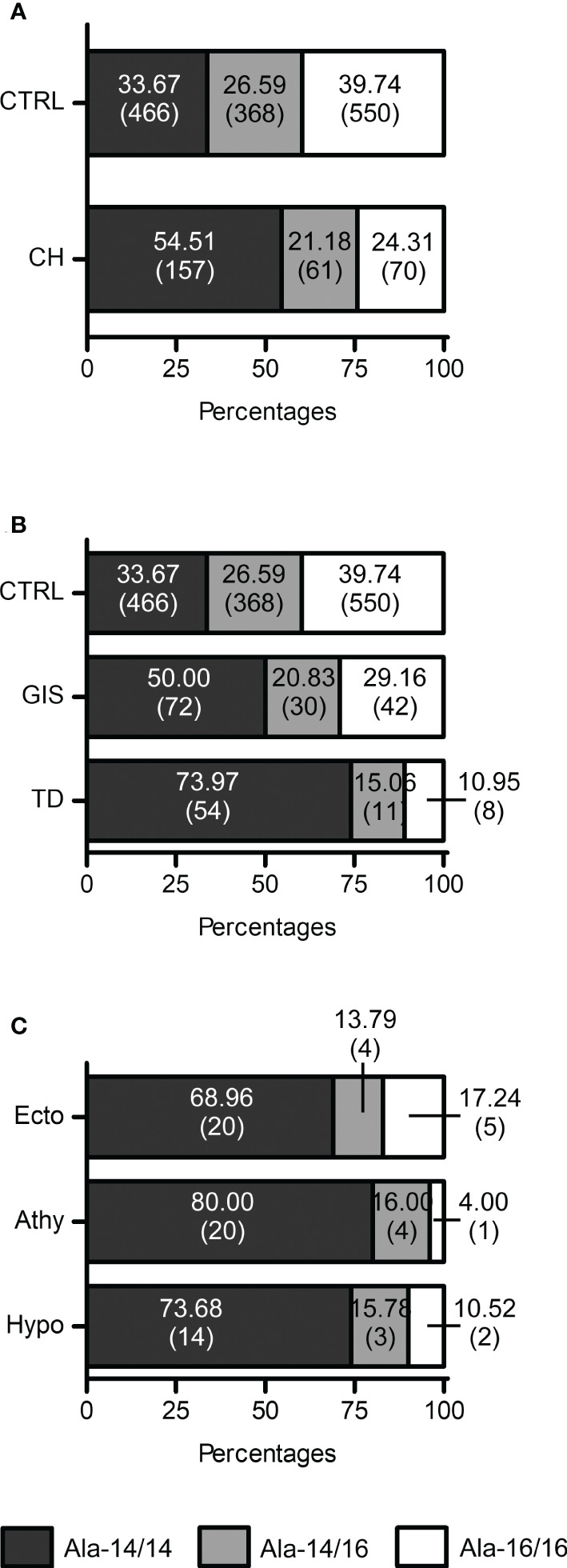
FOXE1 polyalanine genotypes distribution indicates a role of Ala-14/14 in CH and TD. **(A)** Graphical representation of the distribution of the Ala-14/14, Ala-14/16 and Ala16/16 *FOXE1* genotypes among control group (CTRL) (n=1384) and CH cases (n=288); **(B)** graphical representation of *FOXE1* main genotypes distribution among control group (n=1384), GIS (n=144) and TD (n=73) cases. **(C)** graphical representation of FOXE1 main genotypes distribution among TD subgroups (Ecto, ectopy, n=29; Athy, athyreosis n=25, Hypo, hypoplasia, n=19). Contingency was evaluated by Chi-square, Chi-square test for trend and Fisher’s exact test and is reported in the results and discussion section of the article. CH mut, patients with mutations in CH genes.

We identified nine different alleles generating sixteen different combinations of genotypes present in controls and CH groups (NCBI refSNP: rs71369530). In both groups the most frequent alleles were Ala-14 and Ala-16 (**Supplementary Table 2**), but the Ala-14/14 homozygous genotype was predominant in CH patients, either in absolute numbers and in percentages (**Supplementary Table 2**, **Figure 3A**), with a highly significant *X^2^* test for trends (p<0.0001). The allelic combinations different from Ala-14/14, Ala-14/16 and Ala-16/16 represented less than 5% of cases and were not included in further evaluations.

The Ala-14/14 genotype showed a significant association with CH, when compared to Ala-14/16 (odds ratio (OR) 2.031), Ala-16/16 (OR 2.645) as well as to the sum of the latter ones (OR 2.360) (**Table 2**).

**Table 2:** Association of poly-Ala-FOXE1 isoforms with CH and TD.

Polyalanine alleles frequencies in healthy subjects and CH patients					
FOXE1	CH (n)	Healthy Subjects (n)	Fisher’s exact test	Relative Risk (95% CI)	Odds ratio (95% CI)
Ala-14/14	157	466	–	–	–
Ala-14/16	61	368	< 0.0001	1.772 (1.359 to 2.323)	2.031 (1.453 to 2.865)
Ala-16/16	70	550	< 0.0001	2.232 (1.727 to 2.893)	2.645 (1.928 to 3.625)
Ala-14/16 and 16/16	131	918	< 0.0001	2.018 (1.636 to 2.487)	2.360 (1.809 to 3.080)
Polyalanine alleles frequencies in TD and GIS patients					
	TD (n)	GIS (n)	Fisher’s exact test	Relative Risk (95% CI)	Odds ratio (95% CI)
Ala-14/14	54	72	–	–	–
Ala-14/16	11	30	0.096	1.597 (0.970 to 2.821)	2.037 (0.895 to 4.926)
Ala-16/16	8	42	0.0008	2.679 (1.445 to 5.282)	3.909 (1.639 to 10.448)
Ala-14/16 and 16/16	19	72	0.0008	2.053 (1.334 to 3.240)	2.829 (1.479 to 5.580)

CH, congenital hypothyroidism; HS, Healthy Subjects; TD, Thyroid Disgenesys; GIS, gland-in-situ.

Next, we investigated the distribution of the poly-Ala FOXE1 alleles in the different CH subgroups, TD and GIS. Although the Ala-14/14 was the most frequent genotype in both CH subgroups (**Figure 3B**), it was significantly associated with TD (OR 3.909 vs Ala-16/16) (**Table 2**). No significant differences in the poly-Ala tract distribution were detected among the three TD subtypes (**Figure 3C**). At last, among the few patients with FOXE1 heterozygous mutations that we previously identified (7) only the Ala-14/14 genotype was associated with athyreosis (**Supplementary Table 3**).

## 4 Discussion

In this study, we report for the first time that a novel heterozygous point mutation (p.Leu107Val) affecting *FOXE1* DBD may be sufficient to cause thyroid dysgenesis and CH only when associated with homozygous Ala-14-*FOXE1*. The analysis of our large NGS cohort revealed a significant enrichment of the biallelic Ala-14-*FOXE1* genotype in CH, and particularly in TD. In agreement with this data, functional studies showed a significantly impaired transcriptional activity of the p.Leu107Val variant. At variance with the more common Ala-16-*FOXE1*, the 14-Alanine isoform was also found to modify the expression pattern and localization of the transcription factor and to significantly impair the synergic effects that FOXE1 has on the transcriptional activities elicited by NKX2.1 and PAX8.

This study started after the observation that the heterozygous *FOXE1* variant p.Leu107Val segregated with severe CH and athyreosis in 5 siblings of our family (**Figure 1E**). Interestingly, our structural mapping indicates that Leu107 is not in direct contact with DNA. In addition, despite the p.Leu107Val introduces a conservative aminoacidic change in the DBD, we propose that it could modify the interaction with the surrounding hydrophobic regions, indirectly compromising protein stability and affecting its transcriptional functionality (**Figures 1F, G**). All the CH siblings had the same phenotype indicating a minor, if any, pathogenic role for the additional rare variants identified in only one of these patients. The p.Leu107Val variant was inherited from the mother, who presented a gland-*in-situ* with adult-onset nonautoimmune hypothyroidism. This may suggest that, although contributing to alterations in thyroid functionality, this variant is not sufficient *per se* to cause CH. We thus focused on other genetic variants that were present in the 5 affected siblings and found the recurrence of homozygosity for Ala-14 *FOXE1*. This genotype is also present in the euthyroid father, but not in the mother who is carrier of the heterozygous Ala-14/16 *FOXE1.* The co-segregation of an additional non-FOXE1 defect together with the FOXE1 p.L107V variant in all the 5 athyreotic siblings has a low likelihood and this probability would be further lowered if we consider the other two siblings with a CH-like phenotype that died before this study. For these reasons, we propose the concomitant inheritance of the p.L107V variant and the 14-Alanine stretch, together with the CH predisposing background of the family indicated by FOXE1 SNP rs7850258, represents the most likely explanation for the athyreotic phenotype of the 5 siblings.

Variations in the poly-Ala tract length of several nuclear transcription factors emerged late in evolution and were shown to play a fundamental role in their activity (30–32). Accordingly, the poly-Ala tract of FOXE1 is present only in mammals (**Figure 1H**). Interestingly, FOXE1 was reported to affect the transcriptional activity of other thyroid transcription factor, such as NKX2.1 (13, 14). NKX2.1, together with HHEX, PAX8 and FOXE1 strictly regulates in a spatial and temporal manner the complex multiphase process of thyroid development. How these actors interact to finely tune the thyroid function is far to be understood, but they are required for the adequate expression of genes involved in thyrocyte precursors migration, differentiation, proliferation and finally thyroid hormone production (33, 34).

Our data indicate that, although both Ala-14- and Ala-16-*FOXE1* alleles are common in the general population, the two variants have a different modulatory activity on the complex transcription factors network that regulates thyroid development and functionality (**Figure 2D**). From *in silico* structural analysis based on models bound to short DNA fragments, the FOXE1 polyAla predicted helix, although close to the DBD, seems not directly involved in DNA binding (**Figure 1H**), at least considering short DNA linear fragments. Its molecular impact on transcriptional activity, together with that of the large disordered region, is far to be solved at molecular level. Nonetheless, in our cellular studies we report that variations in the poly-Ala length cause alterations in the protein expression and nuclear localization. From here, we hypothesize that the poly-Ala may partially mediate the aggregation or local concentration in the nucleus, by potentially binding and recruiting other transcription factors. These two actions were previously described for other transcription factors containing poly-Ala regions (32, 35, 36) and we propose this might be also the case for FOXE1. Our experiments confirm that functional differences between Ala-16- and Ala-14-FOXE1 become evident and significant only when these isoforms are co-expressed with the other thyroid transcription factors. Notably, when Ala-14-FOXE1 is expressed together with NKX2.1 and/or PAX8, the transcriptional activation of TG promoter is significantly lower than that seen with Ala-16-FOXE1 (**Figure 2D**).

Although the homozygous Ala-14-*FOXE1* genotype significantly increases the risk of TD, as shown by our CH cohort (**Figure 3B**), variations in FOXE1 polyalanine repeats are not sufficient to induce CH *per se*, as around one third of the healthy population has the homozygous Ala-14 tract (**Figure 3A**) (15, 17, 19, 37). Nevertheless, in the few CH patients that have heterozygous FOXE1 variants, athyreosis was present only in the patient with Ala-14/14 genotype, while the Ala-14/16 and 16/16 genotypes were associated with GIS and hypoplasia (**Supplementary Table 3**). Moreover, reports associating *FOXE1* poly-Ala variations with low fT4 levels (38, 39) indicate that the Ala-14 allele may favor the onset of hypothyroidism in combination with other genetic, epigenetic and environmental factors, in the context of a complex origin of CH (6, 7).

In conclusion, the NGS analysis of a large family affected with CH together with the experimental and association studies indicate that homozygous Ala-14-*FOXE1* genotype may contribute to the complex pathogenesis of TD and CH, particularly when combined with heterozygous loss-of-function FOXE1 variant. Therefore, we propose that from now on, the status of *FOXE1* polyalanine tract should be taken in consideration when investigating the genetic origin of CH patients.

Hence, then propose to include *FOXE1* in the group of transcription factors linked to a disease associated with variations of their polyalanine tract (31–33).

Further molecular and cellular studies are needed to elucidate the possible regulatory role of FOXE1 domains other than the DBD and fully understand its role in thyroid development and function.

## Data availability statement

The datasets presented in this study can be found in online repositories. The name of the repository and accession number can be found below: Harvard Dataverse, https://doi.org/10.7910/DVN/KGUPED. Data was collected from human subjects who provided informed consent for its use in the original study, but did not consent for its release to the public. Data access can be required to MD LP (luca.persani@unimi.it), upon reasonable request.

## Ethics statement

The studies involving human participants were reviewed and approved by Istituto Auxologico Italiano. Written informed consent to participate in this study was provided by the participants’ legal guardian/next of kin. Written informed consent was obtained from the individual(s), and minor(s)’ legal guardian/next of kin, for the publication of any potentially identifiable images or data included in this article.

## Author contributions

AG enrolled the CH family and provided clinical and biochemical data. LP conceived and supported the study. DG, EC, and TF performed the bioinformatical analysis of NGS data. EG, GR, SU, TF, MB, and RC-B performed the *in vitro* experiments. FC performed the *in silico* predictions and analysis. EG, GR, and LP interpreted the data; EG, GR, AG, TF, MB, and FC contributed to draft of the manuscript; EG and LP revised the draft and finalized the manuscript. All authors contributed to the article and approved the submitted version.
