# Supplementary material for: The length of FOXE1 polyalanine tract in congenital hypothyroidism: Evidence for a pathogenic role from familial, molecular and cohort studies
Source: Front Endocrinol (Lausanne). 2023 Mar 16;14:1127312. doi: 10.3389/fendo.2023.1127312 (PMC10060985; doi:10.3389/fendo.2023.1127312)
Supplement: Supplementary file 1 [file DataSheet_1.docx]

Supplementary Material

The length of *FOXE1* polyalanine tract in congenital hypothyroidism: evidence for a pathogenic role from familial and cohort studies.

Elisa Stellaria Grassi^1^, Giuditta Rurale^2^, Tiziana de Filippis^2#^, Davide Gentilini^3,4^, Erika Carbone^2^, Francesca Coscia^5^, Sarah Uraghi^6§^, Martyn Bullock^7^, Roderick J Clifton-Bligh^7,8^, Abhinav K Gupta^9^, Luca Persani^1,2^

*^1^ Department of Biotechnology and Translational Medicine, University of Milan, Milan 20122 Italy;*

*^2^ Laboratory of Endocrine and Metabolic Research, IRCCS Istituto Auxologico Italiano, Milan 20149, Italy;*

*^3^ Department of Brain and Behavioral Sciences, University of Pavia, Pavia 27100, Italy*

*^4^ Istituto Auxologico Italiano, IRCCS, Bioinformatics and Statistical Genomics Unit, Milano 20149, Italy*

*^5^ Human Technopole, Viale Rita Levi-Montalcini, 1 - Area MIND – Cargo 6 - 20157 Milano Italy*

*^6^ Department of Health Science, University of Milan, Milan 20122 Italy;*

*^7^ Cancer Genetics Laboratory, Kolling Institute, Faculty of Medicine and Health, The University of Sydney St Leonards, NSW 2065, Australia*

*^8^ Department of Endocrinology, Royal North Shore Hospital, St Leonards, NSW 2065, Australia*

*^9^ Department of Diabetes and Endocrine Sciences, CK Birla Hospitals, Jaipur, Rajasthan, India-302015*

*# now at Synlab Italia, Monza, Italy;*

*§ now at Department of Medicine and Surgery, Milan Bicocca University, Milan 20126, Italy;*

*** Correspondence:** Luca Persani, MD PhD

Laboratory of Endocrine and Metabolic Research, IRCCS Istituto Auxologico Italiano, Milan 20149, Italy; Department of Biotechnology and Translational Medicine, University of Milan, Milan 20122 Italy;

**email:** luca.persani@unimi.it

## Supplementary Figure 1: *in vitro* studies of FOXE1 variants reveal different subcellular localization in HEK cells.


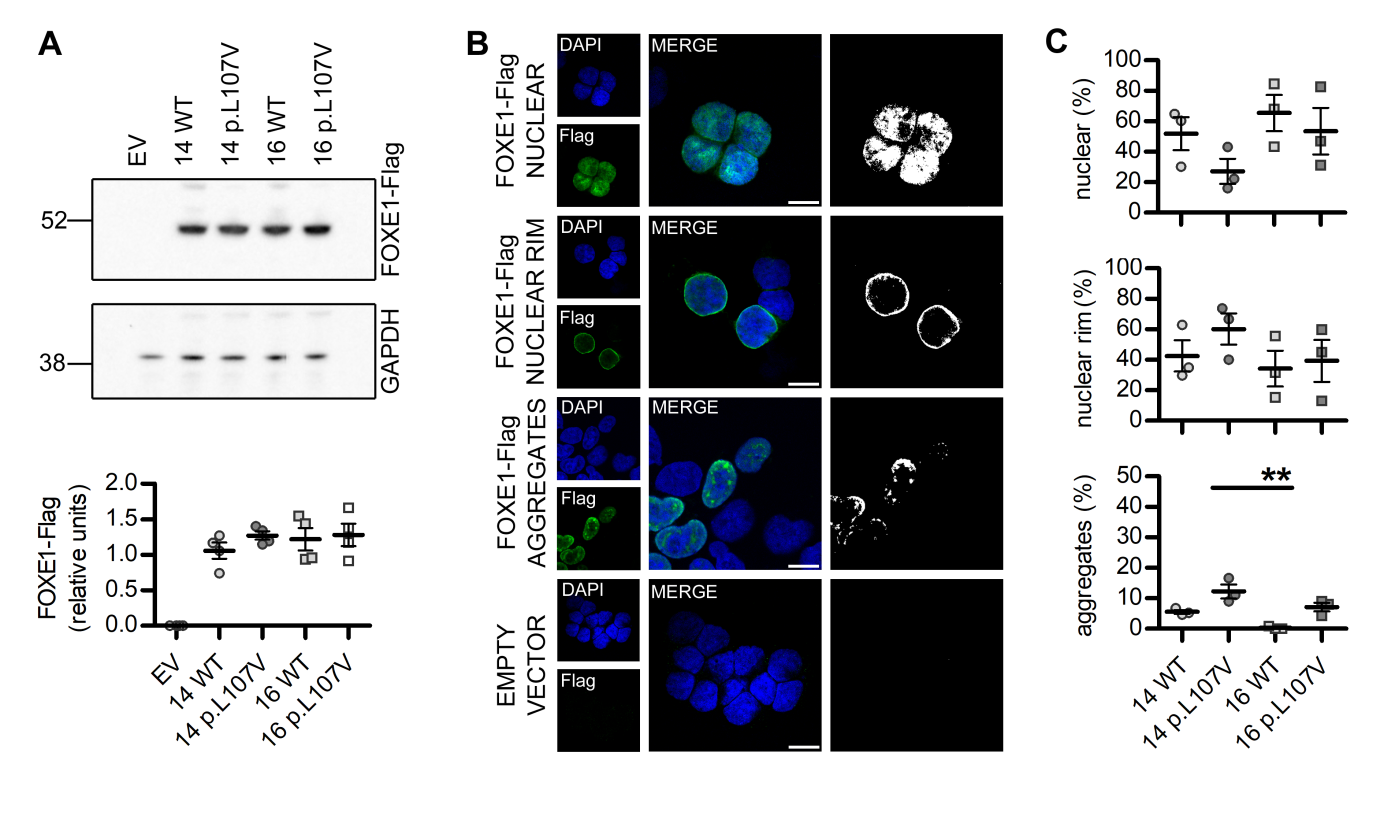


**Supplementary Figure 1.** The (A), representative images and quantification of western blot experiments showing FOXE1 variants expression levels in HEK cells (n=4). (B), confocal microscopy images representative of the different FOXE1 nuclear morphologies, corresponding automatized signal thresholding images and relative quantification of FOXE1 variants nuclear morphologies in HEK cells (n=3; 1796 nuclei analyzed); scalebars 25µm.EV, Empty vector. Statistical significance was determined with one-way ANOVA followed by Bonferroni post-hoc test (A) or Kruskal-Wallis (B). * p<0.05, **p<0.01, ***p<0.001.

## Supplementary Figure 2: *in vitro* studies of FOXE1 variants reveal different subcellular localization – full representative imges


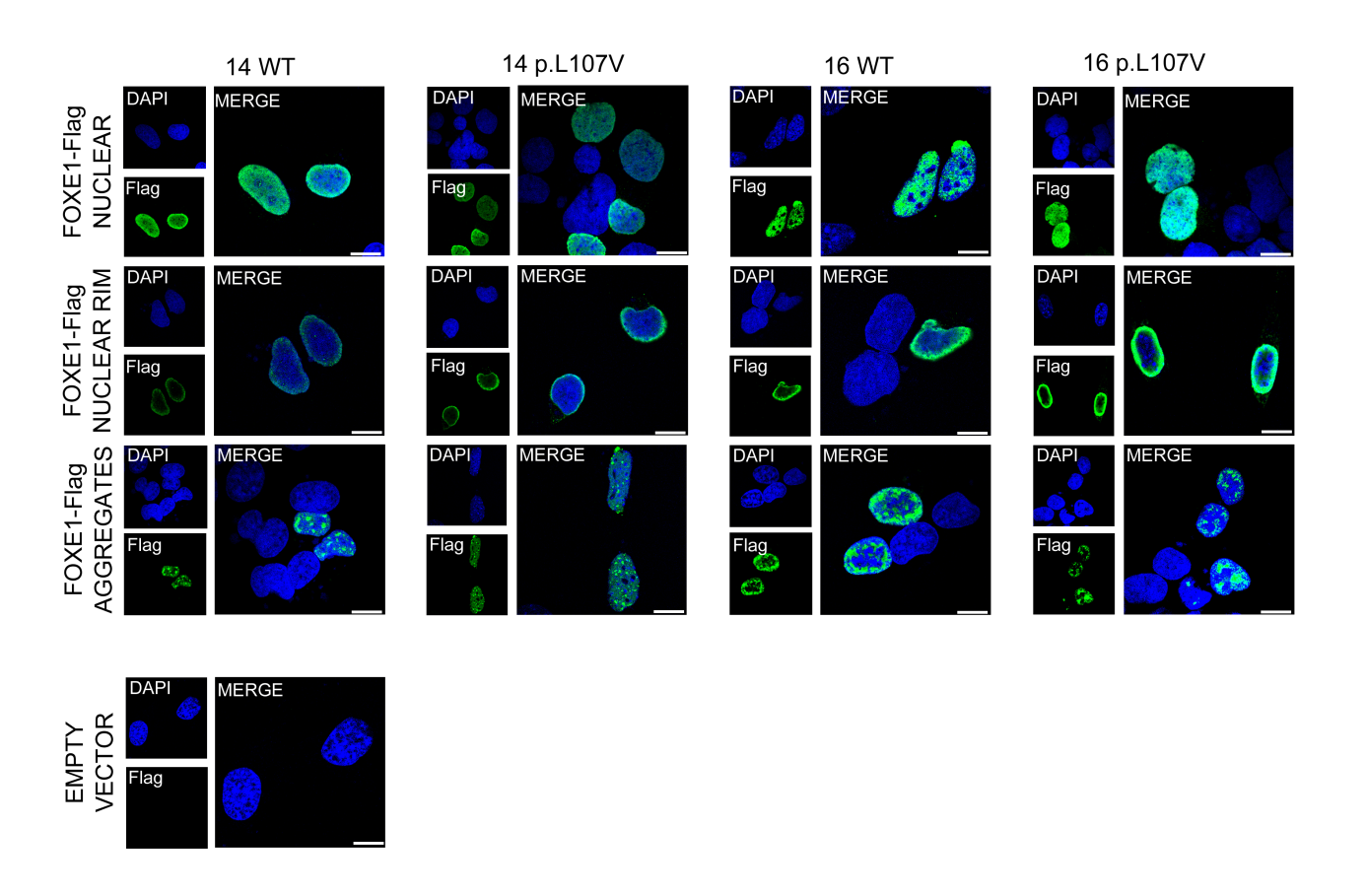
**Supplementary Figure 2:** NTHY-ORI cells confocal microscopy images representative of the different FOXE1 morphologies for each transfected variant.

**Supplementary Table 1: in silico predictions of the variants identified in the family members based on the American the College of Medical Genetics and Genomics (ACMG) recommendation.**

| **Final verdict** | P (PP1+PS3) | LP | B | VUS |
| --- | --- | --- | --- | --- |
| **InterVar Classification** | **LP** (PM1-PM2-PP2-PP3) | **LP** (PM1-PM2-PP3-PP5-BS2) | **B** (BS1-BS2) | **VUS** (PM2-BP4) |
| **Varsome Classification** | **VUS** (PM1+PM2+PP3) | **LP** (PM1+PM2+PP5+PP3+BP1) | **B** (BS1+BS2+BP6+BP4+PP2) | **VUS** (PM2+PP2+BP4) |
| **D out 12** | 11 | 11 | 6 | 0 |
| **ClinVar** | . | Pathogenic | VUS | . |
| **dbSNP** | . | rs121918138 | rs61758083 | rs147583297 |
| **gnomAD_exome_ ALL** | . | 0.0000488 | 0.0049 | 0.00000812 |
| **ExAC_Freq** | . | 0.0000577 | 0.0051 | 0.00000824 |
| **1000G_ALL** | . | 0.0004 | 0.0088 | . |
| **aa position** | p.L107V | p.R101W | p.P135H | p.A518S |
| **Variant** | NC_000009.12(NM_004473.4):c.319C>G | NC_000006.12(NM_203395.3):c.301C>T | NC_000002.12(NM_000547.6):c.404C>A | NC_000019.10(NM_000453.3):c.1552G>T |
| **Gene** | **FOXE1** | **IYD** | **TPO** | **SLC5A5** |

Legend:

BP1 Missense variant in a gene for which primarily truncating variants are known to cause disease.

BP4 Multiple lines of computational evidence suggest no impact on gene or gene product (conservation, evolutionary, splicing impact, etc).

BP6 Reputable source recently reports variant as benign but the evidence is not available to the laboratory to perform an independent evaluation.

BS1 Allele frequency is greater than expected for disorder.

BS2 Observed in a healthy adult individual for a recessive (homozygous), dominant (heterozygous), or X-linked (hemizygous) disorder with full penetrance expected at an early age.PM1 Located in a mutational hot spot and/or critical and well-established functional domain (e.g. active site of an enzyme) without benign variation.

PM2 Absent from controls (or at extremely low frequency if recessive) (see Table 6) in Exome Sequencing Project, 1000 Genomes or ExAC.

PP1 Co-segregation with disease in multiple affected family members in a gene definitively known to cause the disease

PP2 Missense variant in a gene that has a low rate of benign missense variation and where missense variants are a common mechanism of disease.

PP3 Multiple lines of computational evidence support a deleterious effect on the gene or gene product (conservation, evolutionary, splicing impact, etc).

PP5 Reputable source recently reports variant as pathogenic but the evidence is not available to the laboratory to perform an independent evaluation.

PS3 Well-established in vitro or in vivo functional studies supportive of a damaging effect on the gene or gene product.

## Supplementary Table 2: *FOXE1* polyalanine alleles frequencies distribution in the CH and control groups

| Genotypes | CH | | CRTL | |
| --- | --- | --- | --- | --- |
|  | n | % | n | % |
| Ala-10/10 | 0 | 0 | 1 | 0.07 |
| Ala-11/14 | 0 | 0 | 2 | 0.14 |
| Ala-11/16 | 0 | 0 | 1 | 0.07 |
| Ala-12/14 | 2 | 0.68 | 10 | 0.69 |
| Ala-12/16 | 1 | 0.33 | 7 | 0.48 |
| Ala-12/17 | 0 | 0 | 1 | 0.07 |
| Ala-13/13 | 1 | 0.33 | 0 | 0 |
| Ala-13/16 | 0 | 0 | 2 | 0.14 |
| Ala-14/14 | 157 | 52.51 | 466 | 32.07 |
| Ala-14/16 | 61 | 20.41 | 368 | 25.33 |
| Ala-14/17 | 4 | 1.34 | 25 | 1.72 |
| Ala-15/16 | 1 | 0.33 | 1 | 0.07 |
| Ala-16/16 | 70 | 23.41 | 550 | 37.85 |
| Ala-16/17 | 1 | 0.33 | 18 | 1.23 |
| Ala-16/19 | 0 | 0 | 1 | 0.07 |
| Ala-17/19 | 1 | 0.33 | 0 | 0 |

## Supplementary Table 3: Heterozygous FOXE1 point mutations and polyalanine tract length association with thyroid phenotype in CH patients.

| Patient | Thyroid phenotype | Alanine tract | *FOXE1* mutations  **DNA not available**  **DNA not available**  **14/16**  **14/14**  **14/14**  **14/16**  **14/14**  **14/14**  **14/14**  **14/14**  **Pedigree**  ***FOXE1* p.L107V**  **IYD: p.R101W**  **TPO: p.P135H**  **SLC5A5: p.A518S**  **CH**  **CH**  **Healthy**  **CH**  **CH**  **CH**  **CH**  **Healthy**  **Healthy**  **CH**  **CH**  **Healthy** |
| --- | --- | --- | --- |
| P_1 | Athyreosis | 14/14 | p.E34G |
| P_2 | Hypoplasia | 14/16 | p.G30R |
| P_3 | Gland in situ | 14/16 | p.A207V |
| P_4 | Gland in situ | 16/16 | p.Y200X |
